# Toward spreadable entertainment-education: leveraging social influence in online networks

**DOI:** 10.1093/heapro/daz104

**Published:** 2019-10-16

**Authors:** Roel O Lutkenhaus, Jeroen Jansz, Martine P A Bouman

**Affiliations:** 1 Center for Media & Health, Peperstraat 35, 2801 RD Gouda, The Netherlands; 2 Erasmus School of History, Culture and Communication (ESHCC), Erasmus Research Centre for Media, Communication and Culture (ERMeCC), Erasmus University Rotterdam, Burgemeester Oudlaan 50, 3062 PA Rotterdam, The Netherlands

**Keywords:** entertainment-education, social change, spreadable media, transmedia storytelling, audience participation

## Abstract

Entertainment-education (EE) is a communication strategy that uses popular media to engage with audiences on prosocial topics such as health, social tolerance and sustainability. The purpose of EE serials on radio, television or the internet is to introduce new ideas, norms and practices by means of storytelling, as well as to offer points of engagement for audiences to talk about the themes raised by the intervention. However, in today’s media landscape, it has become increasingly difficult to captivate audiences as they have fragmented across channels and have started to create and circulate content themselves. The concept of spreadable media allows us to deal with fragmentation and user-generated content in productive ways, as it recognizes the role of autonomous audience members in shaping the flows of media content in the online networks that underlie today’s media landscape. In this article, we introduce spreadable EE: an innovative approach that builds on transmedia storytelling strategies to reach and captivate target audiences for a longer period of time, and that entails collaboration with online platforms, communities and social influencers to stimulate meaningful conversations. We enhance EE's theoretical, empirical and practical traditions with insights about how today’s audiences have come to engage with media and propose strategic approaches to create and evaluate spreadable EE.

##  

In his lush garden, on a cloudy summer day, we see Bill Gates behind a laptop watching a video of Mark Zuckerberg. Zuckerberg stands next to a bucket of ice-cold water and says some last words before unleashing it onto his head: ‘I'm going to challenge Bill Gates, my partner at Facebook Sheryl Sandberg, and Netflix' founder and CEO Reed Hastings. You have 24 hours to do this, or you have to donate one hundred dollars’.—Splash!

Gates, arms folded, looks up from his laptop. ‘Well, I am glad to accept this challenge, but I want to do it better…’

A bit later, we see Gates on his pier, under a gantry, holding a rope connected to a big bucket of cold water. ‘I’m going to challenge three more people. Elon Musk, Ryan Seacrest, and Chris Anderson of TED, consider yourself challenged!’—Splash! 

In 2014, the ALS Ice Bucket Challenge (Figure 1) was among the first to leverage the power of social influence in online networks, raising $115 million of donations and attention for the national ALS Association—a non-profit organization that seeks to discover treatments and a cure for Amyotrophic Lateral Sclerosis. When celebrities started taking the challenges and started nominating other celebrities, the ALS Ice Bucket Challenge reached unpreceded levels of exposure and engagement, peaking for about 3 months (van der Linden, 2017).

**Fig. 1:**
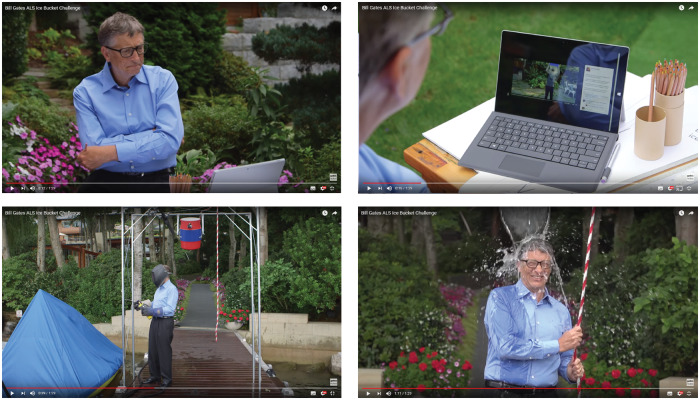
Bill Gates taking the ALS Ice Bucket Challenge (https://www.youtube.com/watch?v=XS6ysDFTbLU).

Over the last decade, health- and social change organizations have experimented with interventions similar to the ALS Ice Bucket Challenge, often with a view to ‘go viral’. But is it right to assume that the ALS Ice Bucket Challenge went ‘viral’? Not according to Jenkins, Ford and Green (Jenkins, 2009, 2013), who argue that ‘going viral’ is a myth. They argue that the virus metaphor implies that media content is capable of spreading itself, infecting one mind after the other as the inevitable result of an irresistible idea, thereby neglecting human agency. Instead, they propose the concept of ‘spreadable media’, postulating that only when appealing media content is meaningfully embedded in the technical infrastructures, economic structures and social networks that underlie the audiences’ media realities, audiences may decide to engage with these ideas autonomously.

From this perspective, the ALS Ice Bucket Challenge did not simply ‘go viral’. Instead, it managed to ‘spread’ because it was well-attuned to the dynamics of the new media landscape. It activated social processes by inviting audiences to participate through a nomination mechanism, gained social momentum by involving a diverse range of celebrities, and translated momentum into real-world contributions through a playful moral imperative (van der Linden, 2017). As such, the ALS Ice Bucket Challenge was intrinsically spreadable.

In this article, we seek to combine lessons learned from a phenomenon like the ALS Ice Bucket Challenge with the entertainment-education (EE) strategy—a communication strategy that uses popular media to spread prosocial ideas. EE typically leverages the appeal of popular media to educate and motivate viewers to improve their health, safety, or equality—mostly using dramatic radio, television and internet serials that allow to engage with a story over a longer period of time (Bouman, 1999, 2016; Singhal and Rogers, 2004; Chatterjee *et al.*, 2017). EE serials apply storytelling to introduce new ideas, norms and practices; and to spark conversations about the issues raised in the serial (Bouman, 1999, 2016; Singhal and Rogers, 2002; Bandura, 2004). As such, EE is not just another message, it is ‘a point of engagement, a site of discourse’ (Storey, 1998). This is important, because—in traditional models of social influence—norms and ideas diffuse through interactions between peers (Rogers, 2003; Katz and Lazarsfeld, 2006). Increasingly, offline societies intertwine with online communities in global digital networks (Bennett and Segerberg, 2012; González-Bailón, 2017)—the same digital networks that enabled the ALS Ice Bucket Challenge to spread. Seeking to leverage the power of social influence in these digital networks, this article enhances EE’s theoretical, empirical, and practical underpinnings and proposes strategic approaches to create and evaluate *spreadable EE*.

## THEORETICAL BACKGROUND

The EE strategy is characterized by an affective approach, using the appeal of popular media on radio or TV to reach target audiences and introduce new knowledge, norms and practices (Bouman, 1999, 2016; Singhal and Rogers, 2004; Chatterjee *et al.*, 2017). It is for good reasons that we find an engaging story at the heart of every EE intervention: stories have always traveled from mouth-to-mouth, eventually settling in cultures and religions as master narratives, which are stories that societies use to make sense of their worlds (Green and Brock, 2005; Halverson, 2011). With their dramatic arcs, stories are capable of captivating audiences over a longer period of time (Branigan, 1992; Green and Brock, 2005).

Narrative theories provide a playground to create compelling and persuasive storylines for EE serials. Studies have shown that stories can be persuasive, capable of impacting individuals’ knowledge, beliefs and attitudes (Green and Brock, 2000). This occurs when audiences are absorbed into a story world where they can identify with the story’s characters—also called *narrative processing* (Slater and Rouner, 2002). Audiences may not only identify with a story’s characters, they may also build imaginary relationships with them: this phenomenon is called *parasocial interaction* (Horton and Wohl, 1956; Papa *et al.*, 2000) and enhances stories’ persuasive effects by negatively affecting the audiences’ capability to critically evaluate messages (Slater and Rouner, 2002). In EE serials, persuasive storylines are often supported by the so-called *heuristic principles*, drawn from Petty and Cacioppo’s Elaboration Likelihood Model (Petty and Cacioppo, 1986; Petty *et al.*, 2005). For example, the *likeability heuristic* implies that audiences tend to place more confidence in people they like—also when these sources are fictional and played by actors.

The EE strategy is also rooted in Albert Bandura’s Social Cognitive Theory (SCT) (Bandura, 1986, 2004). Concepts such as *modeling* and *social learning* contribute to the design of storylines to effectively convey specific ideas, knowledge and practices. Storytelling is also capable of changing the social contexts that shape human behavior. For example, a dramatic storyline about an unplanned pregnancy in a popular TV series can stimulate interpersonal conversations about contraceptives, instilling the uptake of norms that facilitate and support the use of contraceptives (Storey, 1998).

The advantages of persuasive storytelling are apparent, however, not all stories are equally entertaining. Some stories simply stick, whereas other stories are unable to captivate audiences. High-quality storytelling—being in written form, on the radio, or on the screen—is more of an art than a formula (Green and Brock, 2005). The creation of high-quality EE interventions is therefore often a collaborative effort that involves an interdisciplinary team of researchers, health experts, and creative professionals such as scriptwriters, producers and media strategists. The exact nature of these collaborations often depends on the level of involvement of the different partners and shows through the specifics of their partnership agreements (Bouman, 1999; Reinermann *et al.*, 2014).

### Changing media landscape

The media landscape has changed radically since the early nineties, presenting challenges and opportunities for the EE strategy.

First, the media landscapes in Western societies have increasingly saturated through a multiplication of media outlets and options, offering audiences alternative ways to gratify their media-related needs (Sherry, 2002). Audiences often rely on a mix of media and content types to make sense of public issues (Hasebrink and Popp, 2006; Taneja *et al.*, 2012; Kim, 2014; Hasebrink and Hepp, 2017). They have fragmented across platforms to engage with various online communities around specific niche interests, hobbies, or ideologies such as music, sports or politics (Jenkins, 2006; Blank and Reisdorf, 2012). Online communities are characterized by a culture of participation in which members' activities contribute to a collective kind of sense-making (Kligler-Vilenchik and Thorson, 2015).

Second, the introduction of the Internet signifies a shift from the age of the *broadcasting schedule*, where audiences adapt to broadcasting schedules to see their favorite shows, to the age of the *stream*, where audiences choose from a continuous stream of media content at any time they like (Locke, 2016). Conversations in online communities often function as the interface to navigate this stream, meaning that audiences follow up on what peers might ‘like’, share or say on social media sites. Furthermore, online communities often comprise and attract individuals with shared interests and views, increasing the likelihood of audiences confirming their pre-existing beliefs through mutual interactions. This phenomenon is referred to as the *echo chamber* and is often associated with increasing polarization on controversial topics (Colleoni *et al.*, 2014; Barberá et al., 2015b), including health topics such as vaccination (Lutkenhaus *et al.*, 2019b). Moreover, algorithmic recommender systems aggravate this effect: online platforms and social media sites algorithmically personalize their content suggestions to match the supposed media preferences of their users, leading to ‘filter bubbles’ that selectively expose people with similar media patterns to similar content (Pariser, 2012).

Third, some individuals have made a name for themselves in their respective communities and acquired the status of ‘social influencer’ (Langner *et al.*, 2013). Social influencers create their own content and often point their followers to other interesting articles, photos and videos. The role of social influencers is comparable to that of ‘opinion leaders’ in the classic two-step flow model (Katz and Lazarsfeld, 2006) or ‘innovators’ and ‘early adaptors’ in the Diffusion of Innovations Theory (Rogers, 2003). In Katz and Lazarsfeld’s pre-Internet model, mass media would introduce new ideas that flow to opinion leaders who, in turn, would further diffuse these ideas to their peers via interpersonal communication (Katz and Lazarsfeld, 2006). Today, many of these interpersonal conversations take place online where influencers introduce topics, raise questions and spark conversations on a wide variety of issues. As online communities have intertwined with our offline social networks, they play an increasingly important role in the diffusion of ideas, norms and practices in society (Alleyne, 2015; González-Bailón, 2017).

Some have questioned the extent to which online participation can contribute to real life action (Morozov, 2009), while notions such as the 90:9:1 rule (Nielsen, 2006) imply that the part of the audience that actually participates or creates media content is small: 1% ‘heavy contributors’ versus 9% ‘intermittent contributors’ and 90% passive ‘lurkers’. However, it is not just a group of vocal frontrunners shaping the streams of media content. Surrounding the ‘heavy’ and ‘intermittent contributors’, we find large groups of ‘lurkers’ that play a crucial role in amplifying and inhibiting information flows. The media behaviors of this ‘critical periphery’ feed the personalization algorithms with clicks and likes and, in turn, personalization algorithms use these data to determine which media content should be shown, and which not, to whom (Barberá et al., 2015a).

To summarize: changes in the media landscape offer challenges and opportunities to enhance the EE strategy. First, to reach target audiences in an increasingly fragmented and polarized media landscape, there is a need for multi-platforms strategies to align with audience interests to engage with multiple communities at the same time. Second, online communities have emerged as new avenues for audiences to have interpersonal conversations about popular media and EE serials, thereby providing new points of engagement to discuss ideas, knowledge and practices. Third, it has become possible to directly engage with the innovators and early adaptors of online communities via social media influencers. Their key positions in online networks can be leveraged to ‘spread’ new knowledge, ideas and practices, as well as to stimulate, sustain and moderate conversations. In the next section, we will explore how this can be approached in practice, drawing from relevant scientific work and illustrated by practical examples.

## TOWARD SPREADABLE EE

Multi-platform communication strategies can reach audiences that have scattered across the media landscape. In EE, the transmedia storytelling strategy has been used to creatively coordinate elements of a story across platforms, thereby providing multiple entry points across a wide range of channels (Jenkins, 2006; Scolari, 2009; Jenkins *et al.*, 2013). *East Los High* is an example of an EE intervention applying the transmedia storytelling strategy (Wang and Singhal, 2016). This high school teen drama comprises four seasons, running from 2013 until 2017, and is distributed in the US through the video-on-demand platform Hulu. During its first season, the serial focused on sexual and reproductive health among Latina/o Americans. Around the TV serial, online media content provided entry points and more depth to the stories. For example, some characters posted blogs or video dairies, like Ceci—one of the main characters who became pregnant unexpectedly and shared her experiences in a vlog—or Camila—exposing her struggles with her mental health. These stories were often complemented with links to public health services and other reliable information sources, creating pathways between the serial and other layers of relevant content.

The transmedia storytelling strategy can thus be used to reach fragmented audiences by spreading entry points across the platforms and avenues that are popular among their target audiences. Furthermore, the dynamics of social influence in these online communities can be leveraged to stimulate meaningful engagement, such as conversations about EE programs.

### Leveraging social influence

Networks of connected audiences provide the social and technical infrastructure for the circulation of media content (Jenkins *et al.*, 2013) as well as the diffusion of ideas, norms and practices (Katz and Lazarsfeld, 2006; González-Bailón, 2017). Within these networks, communities of like-minded audiences provide avenues to talk about things and topics that interest them, including popular media that may very well include EE serials.

An intervention by the STD/AIDS Foundation in the Netherlands (SAFN) provides an example of how EE professionals can approach online communities as points of engagement. SAFN found that many young Dutch women intend to use condoms, but do not always carry condoms with them because they are afraid to be seen as a ‘slut’. To challenge this norm, SAFN collaborated with social influencers to reach out to online beauty and fashion communities. In a series of YouTube videos (see https://www.youtube.com/watch?v=X2wgJPJguX8), several beauty experts asked their followers for their opinions and, after lively conversations in the comments, summarized them and shared their own opinions. Thereby, SAFN and the social influencers provoked the online communities to challenge the norm from bottom-up, criticizing the idea and ultimately introducing an alternative norm: having condoms with you is smart, not slutty. Eventually, the intervention did not only include influencers sharing SAFN’s message but also invited audiences to reinforce or reappropriate SAFN’s message, ultimately rippling through the social networks around them. As such, SAFN leveraged the dynamics of social influence in these different communities to stimulate meaningful conversations about the topic. 

This example fits well into the theoretical foundations of the EE strategy, where storytelling is a site of discourse that stimulates and sustain meaningful engagement around prosocial topics (Storey, 1998). We will further explore the nature and dynamics of audience engagement in online communities, especially in the context of popular media, and will explore how these dynamics can stimulate audience engagement.

#### Engaging with popular media and narrative exchange

Digital storytelling tools offer audiences rich opportunities to create and share media content of their own (Couldry, 2008; Blank and Reisdorf, 2012). As such, it is often argued that transmedia stories can be expanded by participatory audiences when they create media content relating to the overarching narrative (Jenkins, 2006; Scolari, 2009; Jenkins *et al.*, 2013). When audiences expand a narrative world, they take part in some collective kind of storytelling around a master narrative (Scolari, 2009; Alleyne, 2015) and they add an entry point to the story increasing the EE intervention’s visibility among their networks as a nifty bonus.

Digital storytelling tools can also be used to frame events in a manner that embodies a judgment on their nature (Branigan, 1992). For example, audiences may frame media content in different ways: they can share the same picture, but the captions that they add may imply different meanings and judgments. The process of creating and circulating content around a particular narrative can be understood as ‘narrative exchange’ (Couldry *et al.*, 2014; Clark *et al.*, 2015). The SAFN case shows how audiences can be invited to challenge a norm by engaging in narrative exchange, and how it can contribute to social and behavioral change.

Audience engagement can have a second, more implicit effect, impacting how ideas diffuse and flow through communities. By simply clicking, liking or sharing media content that embody messages or frames that they support, audiences feed personalization algorithms and contribute implicitly to the prevalence of particular frames in the streams of their peers (van Dijck, 2009). As such, members of online communities often engage in a process called ‘networked framing’ (Meraz and Papacharissi, 2013), shaping the course of online conversations.

A common way to conceptualize what happens when ideas spread online is the *meme—*typically a simple image with a caption, often drawn from or making references to popular culture. A ‘meme’ is thought to contain ‘contagious patterns of “cultural information” that get passed from mind to mind and directly generate and shape the mindsets, behavior, and actions of a social group’ [(Knobel and Lankshear, 2007), p. 199]. From a spreadability perspective, we dismiss the idea that ‘memetic content’ is capable of directly generating and shaping mindsets. However, we do acknowledge that a ‘meme’, when making cultural references, can tap into the narrative experiences people have in common, which makes it an effective way of conveying complex messages or ideas using one simple image, especially in the context of storytelling. Plus, it is fairly easy for audiences to create a ‘memetic content’ themselves: it is for good reason that they are used often comments sections. ‘Memetic content’ can play an important role in the conversations EE interventions aim to spur by stimulating the creation of memes with the story’s locations, characters and events as a rewarding source of inspiration. This can be accelerated by referring to community-specific cultures: Kligler-Vilenchik and Thorson found that a *meme* that relates to specific (sub)cultures is more likely to be shared, be imitated, or inspire the creation of new content (Kligler-Vilenchik and Thorson 2015).

#### Setting up story circles to promote narrative exchange

Previous research established that audiences engage in online activities to fulfill needs such as entertainment, finding facts and knowledge, establishing and maintaining social contacts, self-expression, and competition (Shao, 2009; Jansz *et al.*, 2015). Therefore, we cannot assume that target audiences will automatically participate or create content once an EE intervention raises certain issues. For an EE intervention to truly function as a point of engagement, audiences need meaningful incentives to engage in ‘narrative exchange’.

One way to achieve this is by setting up story circles. Clark *et al.* conceptualize ‘story circles’ as ‘a set of agents, processes and infrastructural conditions that enable narratives to consistently emerge and be acknowledged through exchange and mutual interaction’ [(Clark *et al.*, 2015), p. 924]. Clark *et al.* found that, to foster story circles, the technical infrastructure has to be in place and there has to be an incentive to start and sustain narrative exchange, often coming from one or more influential individuals in the network. Moreover, the strongest examples of story circles were the cases in which digital social networks were supplemented by ‘offline’ connections (Couldry *et al.*, 2014). In online communities, the technical infrastructures for ‘story circles’ are in place: the Internet provides platforms where communities of audiences engage with each other. Social influencers and community managers can fulfill the role of ‘story circle’ agents, e.g. by initiating and moderating conversations like the beauty and fashion vloggers did in the earlier mentioned intervention to promote condom use by SAFN. Moreover, EE strategies can draw from narrative persuasion theories and SCT to create innovative media and storytelling formats around social influencers to introduce new ideas, knowledge and practices.

In practice, the key messages of an EE intervention can be layered into a communication strategy to stimulate *narrative exchange* in iterative cycles. For example, in the third season of the Indian EE-series *Main Kuch Bhi Kar Sakti Hoon* (‘I, a woman, can achieve everything’), the social media team of Population Foundation India (PFI) set up story circles around key issues following a four-step cycle: *inspire*, *enable*, *activate*, and *aggregate*. Seeking to promote gender equality, the TV series depicted families celebrating their daughters rather than only their sons (*inspire*). Online, this practice was coined as celebrating *Laadli Din—*a witty combination of the words ‘best’, ‘girl’ and ‘day’*—*providing a label for a practice that can be easily adopted (*enable*). On the show’s Facebook page, audiences were asked to share pictures of their daughters and sisters to celebrate their *Laadli’s* (*activate*), that were combined into new Facebook posts by the community managers to amplify the support for this practice among the audience (*aggregate)*. This led to a series of posts with audiences sharing their interpretations of *Laadli Din* and comments about the role of girls and women in the family challenging existing gender regressive norms.

#### Markers

The word ‘*Laadli Din*’ provides audiences with a new and uniquely labeled behavior that can be easily adopted. In EE, such a specific word or practice is also known as a *marker*. Markers are unique identifiable elements of messages such as new words, phrases or novel behaviors that ideally model new realities to break oppressive power structures in society (Singhal and Rogers, 2002; Bouman *et al.*, 2012; Wang and Singhal, 2018). The goal of markers is two-fold: through uptake, markers directly contribute to attaining EE interventions’ goals, while also enabling researchers to track conversations around the marker for monitoring or evaluation purposes. The latter solves an important research issue: any marker-related online activity can now be directly attributed to the EE intervention as a result of the marker’s uniqueness. For example, the Center for Media & Health collaborated with the Dutch daily soap ‘Good Times, Bad Times’ to introduce the markers ‘haperhoofd’ (Dutch for ‘stuttering head’, referring to cognitive malfunction resulting from brain damage) and ‘cocakop’ (Dutch for ‘cocaine head’, referring to somebody with a cocaine addiction), tracked conversations around these words by scanning social media platforms, and analysed the audience’s responses (Bouman *et al.*, 2012). 

In the digital age, markers do not necessarily have to be words: we can also think of other forms and modalities that are easily replicable in text, photos or videos such as symbols, gestures or dance moves. Markers can even include digital stickers, animations or augmented reality via Facebook Filters, Frames or Snapchat Effects, appealing to the playfulness of the target audiences. By including stickers, GIFS and visual effects that only refer to particular scenes, characters and events (e.g. *Laadli Din*), a visual lexicon of markers may shape the course a conversation takes. Similarly, *East Los High* provided easily sharable content such as healthy recipes and dance routines drawn from the TV show, promoting conversations about healthy food and exercise.

To conclude, an important advantage of markers is that we can let audiences reaffirm markers from bottom up, meaning that they can use digital storytelling tools to reaffirm and recontextualize markers to reflect their own realities. As these recontextualized markers diffuse through digital networks, they are enriched with various stories and real-world experiences and empower audiences to have a meaningful conversation about the topics, themes, or issues that resonate with them most strongly—closing the loop from bottom-up.

### Research and evaluation

Research and evaluation play an important role in the field of EE, and it is critical to position *spreadable EE* within the field’s rich research tradition. EE distinguishes between formative research, which is applied to inform the design of an intervention, and summative research to measure the intervention’s effects (Bouman, 1999). Today, it is possible to leverage public data sources for formative research from platforms like Twitter, YouTube and Facebook to retrieve information on how communities of audiences are connected, how they talk about certain themes and issues, and which individuals are among the most influential (Lutkenhaus *et al.*, 2019a). Such research methods are essential to identify target audiences and to strategically decide on which influencers to collaborate with.

Likewise, the analysis of online communities, conversations and social influence can be used for summative research and contribute to the evaluation of the intervention, e.g. by monitoring how conversations change over time or tracking the diffusion of markers. Digital methods provide tools to study the behaviors and dynamics of online communities and play a critical role in the evaluation of *spreadable EE* interventions. EE professionals need to collaborate with community managers and data scientists to bring this into practice.

### Collaboration

The field of EE has a long-standing tradition of interdisciplinary collaboration. During the late nineties, Bouman (Bouman, 1999, 2002) studied strategies for EE collaboration in television formats between health communication professionals and media professionals. Bouman found that if different professional domains want to collaborate, they have to have a feel for the game and know the *habitus* of each other’s fields. The same is true for *spreadable EE*, although the stakeholders are different. Depending on the scope and context, *spreadable EE* requires collaboration with a new kind of media professionals such as social influencers, content strategists and data analysts. These professionals have unique professional and educational backgrounds and EE professionals need to be acquainted with what these new stakeholders bring to the table in order to work toward a common frame of reference.

## DISCUSSION

The significance of our contribution is that it reevaluates the EE strategy in the light of changes in the media landscape such as media saturation, audience fragmentation and algorithmic personalization. Seeking to leverage social influence in digital networks, it expands existing EE theories with insights and strategies from the new media landscape and proposes approaches to create *spreadable EE* in practice. As such, *spreadable EE* utilizes the dynamics of media engagement and social influence in digital networks to create sites of engagement where audiences can discuss new ideas, knowledge, and practices, while empowering audiences to highlight the aspects that matter to them the most.

A limitation is that we have described interventions that vary in scale and scope, while the specifics of EE strategies usually are a matter of goals, budgets and other contextual realities. The skills, expertise and collaboration partners needed to create *spreadable EE* vary largely as well. Nonetheless, we have discussed the main practical implications of *spreadable EE*, such as leveraging digital methods for formative and summative analysis and working toward interdisciplinary collaborations. Future studies could further explore methodological innovations and the dynamics of interdisciplinary collaborations in *spreadable EE*.

Furthermore, it is often assumed that health- and social change organization possess too little resources to compete with vested industries that are marketing unhealthy products such as tobacco, alcohol and fast food; promoting unsustainable products such as cars, single-use plastics and clothing; and creating entertainment media showing irresponsible and intolerant behaviors. Compared to health- and social change organizations, vested industries possess more resources to generate clicks, views and likes through paid adverting and other outreach strategies. The power of *spreadable EE* lies not in reach, but in the quality of engagement of specific target audiences with the EE intervention, as these actions will ripple through their social networks. In this context, EE professionals play the role of conductors, orchestrating a ‘transmedia symphony’ (Gomez, 2010) that sheds light on all relevant aspects of social issues, and empowers audiences to join in and share their perspectives.

When it comes to stimulating conversations about prosocial topics, maintaining in control over a *spreadable EE* intervention is a delicate matter. *Narrative exchange* may quickly take alleys that health communicators might want to avoid, like the Kony 2012 case that faced this backlash when communities of audiences started to create *memetic content* accusing the campaigns’ supporters of slacktivism (von Engelhardt and Jansz, 2014; Kligler-Vilenchik and Thorson, 2015). The Kony example shows that EE also risks being subverted, that its social momentum can be taken hostage by a different group that uses it to flip the message. This lack of control is a characteristic typical for the dynamics in the networks of connected audiences that underlie the media landscape today (Rainie and Wellman, 2012). Health- and social change organizations should embrace the dynamic nature of the internet by approaching *spreadable EE* like an ongoing conversation. For example, instead of repressing backlash, EE professionals could respond to concerns or use it as input for a public discussion amongst the audience.

## CONCLUSION

In this article, we have shared our perspective on the premise of *spreadable EE*, illustrated by theoretical notions and practical examples. *Spreadable EE* is built upon transmedia storytelling strategies that foster audience participation and effectively reach audiences that have spread across the media landscape. Persuasive storytelling strategies keep audiences engaged over a sustained period of time, and audience engagement is stimulated by setting up *story circles.* There, social influencers introduce new ideas, knowledge and practices, and stimulate conversations around prosocial topics. Narrative elements and multi-modal markers provide means to shape the course of *narrative engagement* and yet empower audiences to reaffirm and recontextualize markers to reflect their own realities. Furthermore, the use of markers allows EE professionals to follow conversations around key concepts of particular EE interventions in order to track the diffusion of ideas, knowledge and practices.

## FUNDING

This work is supported by a grant from the Dutch Friends Lottery (MediaLab Project). 
